# Seasonal and Spatial Variations of the Oxidative Properties of Ambient PM_2.5_ in the Po Valley, Italy, before and during COVID-19 Lockdown Restrictions

**DOI:** 10.3390/ijerph20031797

**Published:** 2023-01-18

**Authors:** Maria Chiara Pietrogrande, Cristina Colombi, Eleonora Cuccia, Umberto Dal Santo, Luisa Romanato

**Affiliations:** 1 Department of Chemical, Pharmaceutical and Agricultural Sciences, University of Ferrara, Via Fossato di Mortara 17/19, 44121 Ferrara, Italy; 2 Environmental Monitoring Sector, Arpa Lombardia, Via Rosellini 17, 20124 Milano, Italy

**Keywords:** PM_2.5_ oxidative potential, dithiothreitol and ascorbic acid cell-free assays, chemical tracers, Po Valley, seasonal and spatial variations, Coronavirus pandemic, lockdown restrictions

## Abstract

This study describes the chemical and toxicological characteristics of fine particulate matter (PM_2.5_) in the Po Valley, one of the largest and most polluted areas in Europe. The investigated samples were collected in the metropolitan area of Milan during the epidemic lockdown and their toxicity was evaluated by the oxidative potential (OP), measured using ascorbic acid (OP^AA^) and dithiothreitol (OP^DTT^) acellular assays. The study was also extended to PM_2.5_ samples collected at different sites in the Po Valley in 2019, to represent the baseline conditions in the area. Univariate correlations were applied to the whole dataset to link the OP responses with the concentrations of the major chemical markers of vehicular and biomass burning emissions. Of the two assays, OP^AA^ was found mainly sensitive towards transition metals released from vehicular traffic, while OP^DTT^ towards the PM carbonaceous components. The impact of the controlling lockdown restrictions on PM_2.5_ oxidative properties was estimated by comparing the OP values in corresponding time spans in 2020 and 2019. We found that during the full lockdown the OP^AA^ values decreased to 80–86% with respect to the OP data in other urban sites in the area, while the OP^DTT^ values remained nearly constant.

## 1. Introduction

Recently, many researchers reported that the lockdown measures imposed for the COVID-19 pandemic control generated an improvement in air quality [1,2,3,4,5,6,7,8,9]. This has been mainly associated with the reduction in some anthropogenic emissions, attributed mostly to road traffic and less to industrial activities, as a consequence of limitations of most human activities. Thus, the COVID-19 shutdowns around the world have provided a unique opportunity for researchers to estimate the impact of significantly reduced anthropogenic emissions on the chemical characteristics of ambient air. Among the air contaminants, fine particle matter (PM_2.5_) is of great importance due to its distinct physio-chemical characteristics and well-established adverse health consequences, e.g., respiratory and cardiovascular disease. Among the possible toxicological mechanisms of PM-induced health effects, there is increasing consensus on the involvement of oxidative stress caused by excessive accumulation of reactive oxygen species in the human body [10,11]. Thus, the oxidative potential (OP), representing the capacity of PM to oxidize molecules generating reactive oxygen species (ROS), has been suggested as a representative metrics of PM toxicity related to oxidative stress [12,13,14,15,16,17].

Although the influence of the epidemic restrictions on the air quality has been described in a great number of papers [1,2,3,4,5,6,7,8,9,18,19,20,21,22,23,24], to our knowledge, the impact on aerosol toxicity has been reported only in a few papers [2,6,22,23]. Therefore, the main goal of this work was to investigate the influence of quarantine lockdown on the oxidative toxicity and chemical composition of PM_2.5_ particles to understand the change of PM properties before and during the pandemic shutdown. In this context, the study of the Po Valley in Italy appears particularly representative, since it has been the epicenter of COVID-19 in Europe, with unmatched rates of confirmed infectious individuals and lethality. Thus, Italy was the first western country to apply severe measures, including a general lockdown, with most of the population confined at home and a shutdown of all nonessential productive activities and services [4,7,9,18,19,20,21,24]. The Po Valley is regarded as an air pollution hot spot in Europe, with frequent severe PM pollution events linked to the heavy impact of anthropogenic emissions—from industry, agriculture, traffic, burning emissions—as well as of high photochemical activity [25,26,27,28,29].

We measured the chemical oxidative potential and chemical composition of fine particles collected at a traffic site in the metropolitan area of Milan for 5 months in 2020, encompassing different phases of the lockdown restrictions. Oxidative potential was assessed by using acellular assays, since they have the advantages of faster reading speed, lower price, less controlled environments and suitability for automation, in comparison with cellular assays [17,30,31,32,33]. They are based on target antioxidants miming PM–cell interactions, such as dithiothreitol (DTT), as a proxy of cellular reductants [15,34,35], and ascorbic acid (AA), as a chemical surrogate of physiological antioxidants in the respiratory tract lining fluids [11,36,37,38]. The impact of the lockdown restrictions on PM oxidative properties was estimated by comparison with the OP responses measured from PM_2.5_ filters collected in the Po Valley from March 2018 to February 2020, to describe baseline conditions in the area. These data were retrieved from our previous studies at urban and rural sites in the Lombardia and Emilia Romagna regions, located in different parts of the Po Valley [39,40,41]. For each investigated site, the PM_2.5_ oxidative properties were reported and their correlations with the concentrations of the main chemical markers were computed to highlight the PM redox-active components driving OP. This study enabled us to single out the impact of reduced anthropogenic emissions, mainly from vehicular traffic, on PM_2.5_ oxidative toxicity in a region characterized by alarming PM conditions. In addition, this work gives a deeper insight into the lockdown effects on the toxicity of PM particles in the metropolitan area of Milan, previously described by Altuwayjiri [2] for PM_2.5_ and by the Authors for the PM_10_ fraction [23].

## 2. Materials and Methods

### 2.1. Chemicals and Materials

Monosodium phosphate (NaH_2_PO_4_) and disodium hydrogen phosphate (Na_2_HPO_4_) were purchased from Fisher Scientific (Rodano, Milan, Italy). They were used to prepare 0.1 M phosphate buffer at pH 7.4 with ultrapure water (resistivity = 18.2 M Ω⋅cm) obtained in a Milli-Q^®^ IQ 7000 water purification system (Merck KGaA, Darmstadt, Germany). Then, the buffer was eluted through a Chelex^®^ 100 sodium form resin (Bio-Rad, Segrate, Milan, Italy) to remove any metal contamination.

Solutions of DTT and DTNB (5,5′-dithiobis(2-nitrobenzoic acid)) (Sigma Aldrich s.r.l., Milan, Italy) were prepared in phosphate buffer (10 mM), while solutions of L-ascorbic acid sodium salt (AA) (Sigma Aldrich s.r.l., Milan, Italy) were prepared in ultrapure water (10 mM). Aqueous solutions of the reagents were preserved in amber glass vials in the dark at −20 °C, since they are unstable at room temperature and sensitive to light.

### 2.2. Sampling Site and Periods

**Figure 1:**
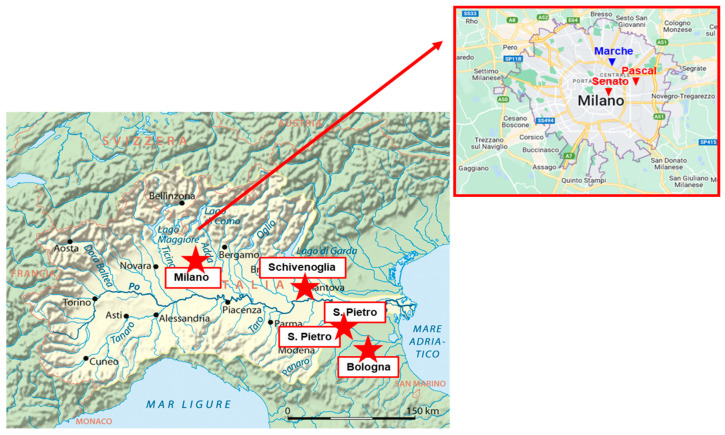
Map of the Po Valley study area (https://geography.name/po-valley/ accessed on 2 December 2022); enlarged insert: map of the metropolitan area of Milan (©2019 Google). Symbols indicate the locations of all sampling sites.

Sampling was conducted inside the metropolitan area of Milan, a city with 1.4 million inhabitants, at the Marche site of the ARPA Lombardia Air Quality Network. Marche is an urban traffic station located in the northeastern part of the external ring road (Lat 45°29′46.76″ N, Long 9°11′27.43″ E) impacted by heavy traffic (location indicated by a blue star in the enlarged detail of Figure 1). Daily PM_2.5_ samples were collected from 2 January to 26 May 2020, encompassing different phases of the COVID-19 national lockdown: (1) pre-lockdown (preL, 2 January–25 February) with normal conditions; (2) first partial lockdown (PL1, 26 February–24 March) when schools and universities were closed, public and religious events were cancelled and movement was limited in the Po Valley; (3) full lockdown (FL, 25 March–4 May) imposing drastic restrictive measures limiting travel, social, cultural and economic activities, with most of the population confined at home and a shutdown of all nonessential productive activities and services; (4) second partial lockdown (PL2, 5–18 May) with progressive limitations relaxation, as most of the indoor and outdoor activities were re-opened and free movement between regions was allowed.

For each campaign day, PM_10_ and PM_2.5_ particles were sampled in parallel on 47 mm diameter Teflon (Pall) and quartz microfiber (Pall) filters (47 mm diameter). A low volume automatic outdoor sampler (Skypost PM, TCR-TECORA, Cogliate (MB), Italy) was used, operating at the standard air flow rate of 38.3 L min^−1^ for 24 h to collect an air volume of ≈55 m^3^ per day [23,41]. The PM_10_ and PM_2.5_ mass concentrations were determined on Teflon filters, at 50% relative humidity and 20 °C, following the UNI-EN12341 procedure [42].

### 2.3. Chemical Characterization

Particle matter chemical analyses were performed in the laboratories of the Environmental Monitoring Sector, Arpa Lombardia. Details are reported elsewhere [41]. Briefly, the elemental composition of each PM_10_ and PM_2.5_ sample was determined by energy dispersive X-ray fluorescence (ED-XRF). An Epsilon 4 spectrometer was used from Malvern Panalytical (Monza, Italy). Four measuring conditions were chosen to optimize the sensitivity for the 18 analytes, i.e., Al, Si, P, S, Cl, K, Ca, Ti, V, Cr, Mn, Fe, Ni, Cu, Zn, Br, Pb, Rb. On the PM_10_ quartz filters, the mass concentrations of elemental (EC) and organic carbon (OC) were measured with the TOT/TOR technique using a SUNSET EC/OC instrument (Sunset Laboratory Inc., Tigard, OR), according to the NIOSH-like and EUSAAR-2 protocols. Several anions, cations and sugars were also quantified from each PM_10_ quartz filter; in particular, anhydrous sugars were determined using ion-chromatography, IC equipped with an amperometric detector.

### 2.4. Assessment of the PM Oxidative Potential

The OP was quantified on each PM_2.5_ Teflon filter using the DTT and AA assays, following the procedure described elsewhere [31,41,43]. Briefly, in both assays, a known amount of the target antioxidant AA or DTT (30 µL of a 10 mM AA or DTT solution) was added to the water extract of a filter quarter and their concentrations were measured by using a UV-Vis spectrophotometer (Jasco V-730, Jasco Europe s.r.l., Lecco, Italy). The oxidative potential is expressed as the depletion rate (nmol min^−1^) of DTT or AA, which is proportional to the generation rate of ROS. It was computed by linearly fitting the experimental points of the reagent concentration versus time (5, 10, 15, 20, 30 min) [31,43]. The response of blank filters was determined and subtracted from the data of real PM_2.5_ samples. The obtained OP responses were normalized to the volume of the sampled air, to obtain an exposure metrics accounting for inhaled air (OP_V_, nmol min^−1^ m^−3^), and for the mass of the sampled particles, to compute a parameter describing the PM intrinsic oxidative properties (OP_m_, nmol min^−1^ µg^−1^).

### 2.5. Statistical Analysis

The data were analyzed by descriptive statistics and reported as mean ± standard deviation. Two-tail *t*-test was applied to identify significance between the means of the values in the different investigated periods. Pearson’s correlation coefficient was used to investigate the association of the OP responses with the PM_2.5_ chemical components. A *p* value ≤ 0.01 was considered as the significant level.

## 3. Results

### 3.1. PM_2.5_ Oxidative Potential during Lockdown Periods

**Figure 2:**
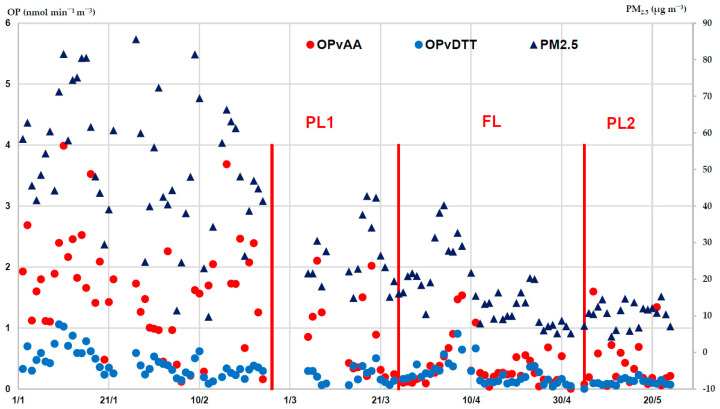
Temporal evolution along the whole investigated time of the extrinsic volume-based OP^AA^_V_ (red points, left Y scale) and OP^DTT^_V_ (light blue points, right Y scale) responses and PM_2.5_ concentrations (blue triangles, right Y scale). The vertical lines indicate the first day of each lockdown period, namely 26 February for PL1, 25 March for FL and 5 May for PL2.

The OP^AA^ and OP^DTT^ responses were measured for each PM_2.5_ filter and their temporal evolution was investigated, with specific concern to the different lockdown periods. The volume-based responses OP^AA^_V_ (red points, left Y scale) and OP^DTT^_V_ (light blue points, right Y scale) are presented in Figure 2, together with the measured PM_2.5_ concentrations (blue triangles, right Y scale), with the vertical lines indicating the PL1, FL and PL2 periods.

A visual inspection of the data clearly shows a similar trend of both AA and DTT responses, as well as for the PM_2.5_ mass concentration, with a strong reduction from 26 February, when the first partial lockdown started. This trend was also investigated by applying the *t*-test to the mean and standard deviation values computed for each lockdown period (Table 1). The significant (at *p* ≤ 0.05 level) changes between the periods were identified (indicated by * in the table). In particular, the largest effect was shown from preL to FL on the OP^AA^_V_ responses, which decreased by nearly 4.2 times, from 1.72 ± 0.78 to 0.38 ± 0.37 nmol min^−1^ m^−3^, while the OP^DTT^_V_ was nearly halved, from 0.43 ± 0.22 to 0.25 ± 0.20 nmol min^−^1 m^−3^, following a similar trend as PM_2.5_ mass, from 51.46 ± 18.43 µg m^−3^ to 15.03 ± 7.42 µg m^−3^. Then, the decreased values remained almost constant in the subsequent PL2 period for the three parameters. The PM_2.5_ chemical compositions were characterized based on the main chemical tracers, in order to investigate the possible contribution to the PM_2.5_ redox properties of emission sources and atmospheric processes. The concentrations of 18 major and trace elements were measured for each sample and the average and standard deviation values were computed for each lookdown period (Table 1). Lacking experimental data of the PM_2.5_ organic components, the concentrations of OC and levoglucosan, a tracer of wood combustion, were estimated from the measurements on the PM_10_ samples collected in parallel (values reported in italics in Table 1). A conversion factor was used on the basis of the wide dataset of PM measurements carried out in the laboratories of the Environmental Monitoring Sector, Arpa Lombardia [12,20,21,23,41,44,45]. Overall, OC was the most abundant component, with concentration mean ≥ 1 µg m^−3^, in particular in winter. Of the other species, the most abundant were elements S and Fe (concentration mean ≥ 500 ng m^−3^), followed by other inorganic components (at concentration levels ≤ 300 ng m^−3^) − Si, K and Ca − and trace metals (at concentration levels ≤ 50 ng m^−3^). The Student’s *t*-test (at the significance level α ≤ 0.05) singled out a significant decrease from the normal PreL to the lockdown periods for most of the determined components, namely OC, levoglucosan, P, S, Cl, K and the heavy metals, i.e., Cr, Mn, Fe, Cu, Zn and Pb, following the general decrease in PM_2.5_ mass concentration (values marked by * in Table 1).

**Figure 3:**
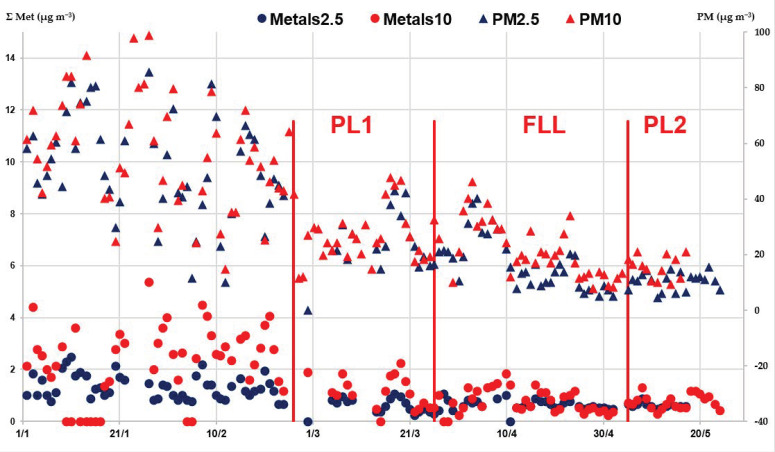
Temporal evolution along the whole investigated time of the total sum of the concentrations of traffic-related metals (Σ metals) in PM_2.5_ (blue points, left Y scale) and PM_10_ fractions (red points, left Y scale) and PM_2.5_ (blue triangles, right Y scale) and PM_10_ mass concentrations (red triangles, right Y scale). The vertical lines indicate the first day of each lockdown period, namely 26 February for PL1, 25 March for FL and 5 May for PL2.

The concentrations of redox-active metals were also measured in the PM_10_ filters simultaneously collected in the study campaign, with the aim of investigating their PM size distribution. The total sum (Σ metals) was computed for the most relevant traffic-related metals, i.e., Cr, Mn, Fe, Ni, Cu, Zn and Pb (Table 1). The temporal evolution of Σ metal concentrations in both fractions was evaluated along the whole investigated time, together with those of PM_2.5_ and PM_10_ mass concentrations (Figure 3). The obtained results showed the prevalent metal accumulation in the PM_10_ particles, mainly during the PreL and Pl1periods, with nearly double concentrations than the fine PM, i.e., 2.71 ± 0.91 µg m^−3^ and 1.13 ± 0.36 µg m^−3^ in comparison with 1.29 ± 0.49 µg m^−3^ and 0.62 ± 0.26 µg m^−3^ in PreL and PL1, respectively.

Then, to give insight into the contribution of these PM_2.5_ components to the particle OP, we investigated their association with AA and DTT activity. Indeed, only some of the selected species were found to be reactive towards the OP assays—redox-active metals (e.g., Mn, Fe, Cu, Zn)—while the others correlated or inter-correlated with them [31,32,38,43,46,47]. Based on Pearson’s correlation coefficient, most of the investigated species showed significant association with the OP_V_ responses (*p* ≤ 0.01, values in bold in Table 2), with only small differences between the OP^AA^ and OP^DTT^ values. In detail, both OP^AA^_V_ and OP^DTT^_V_ responses were significantly correlated with the carbonaceous components, OC, levoglucosan, elements Cl, K and Br and some transition metals—Cr (only OP^AA^_V_), Mn, Fe, Cu, Zn and Pb.

### 3.2. Overview of PM_2.5_ Oxidative Potential in Po Valley

To investigate the impact of the lockdown restrictions on PM_2.5_ oxidative properties we need OP data describing the baseline conditions commonly present in the investigated area. Attention must be paid in comparing OP measurements, since they are widely assay- and location-dependent and scarcely available from the literature [30,31,32,39]. Thus, to assure the best data comparability, this study was based on OP responses previously measured in our laboratory [23,39,40,41]. The data concern PM_2.5_ filters collected during various seasons from March 2018 to February 2020 in different sites in the Po Valley, giving an overview of PM_2.5_ OP in the region (Table 3). The map in Figure 1 gives a general insight into the locations of all sampling sites across the Po Valley, while the enlarged inset describes the metropolitan area of Milan. Two study locations are in the Lombardia region, as included in the ARPA Lombardia Air Quality Network [29]. They are Milano Pascal, which is an urban background station located on the eastern side of Milan, and Schivenoglia, which is a rural background station, located in the southeastern part of the Lombardia region, far from specific pollution sources. The other two investigated locations are in the Emilia Romagna region, in the south-eastern part of the Po Valley. One is an urban background site located in the center of the city of Bologna (~400,000 inhabitants), and the other a rural background station located at San Pietro Capofiume, about 30 km northeast of the city.

In order to account for the strong variability with seasons and atmospheric conditions, the reported data were grouped according to the time spans corresponding to the lockdown periods in 2020, namely winter (PreL), spring (Pl1 and FL) and early summer (PL2). For each period, the mean values were computed for the oxidative responses and the concentrations of PM_2.5_ mass and the main source markers. i.e., organic carbon (OC), levoglucosan and the sum of traffic-related metals (Table 4). When the experimental measurements were not available, some OC and levoglucosan data were estimated from the values determined on the PM_10_ filters collected in parallel (values in italics in Table 4) [24]. The Student’s t test was conducted to single out significant differences among the data (signified by * in Table 4).

First of all, we can observe that both OP^AA^ and OP^DTT^ metrics show a common pattern for all the sites across the Po Valley, characterized by higher OP^AA^ than OP^DTT^ responses, a clear seasonality—with increased values in winter compared with warm seasons—and small differences among the sites for each investigated season. This last characteristic may be explained by varying emission sources, but homogenous meteorological conditions all over the region, at least as described by the close value ranges for temperature and solar radiation in each investigated season, namely 6–9 °C, 10–19 °C, 19–24 °C for temperature and 170–220 W m^−2^, 240–300 W m^−2^ and 80–110 W m^−2^ for solar radiation in winter, spring and early summer, respectively (Table 4). This may also be associated with homogenous PM_2.5_ mass concentrations among the sites, which ranged between 34 and 51 µg m^−3^ in winter, from 16 to 26 µg m^−3^ in April and from 10 to 15 µg m^−3^ in early summer (Table 1 and Table 4).

### 3.3. Association of PM_2.5_ Components to Oxidative Potential in Po Valley

**Figure 4:**
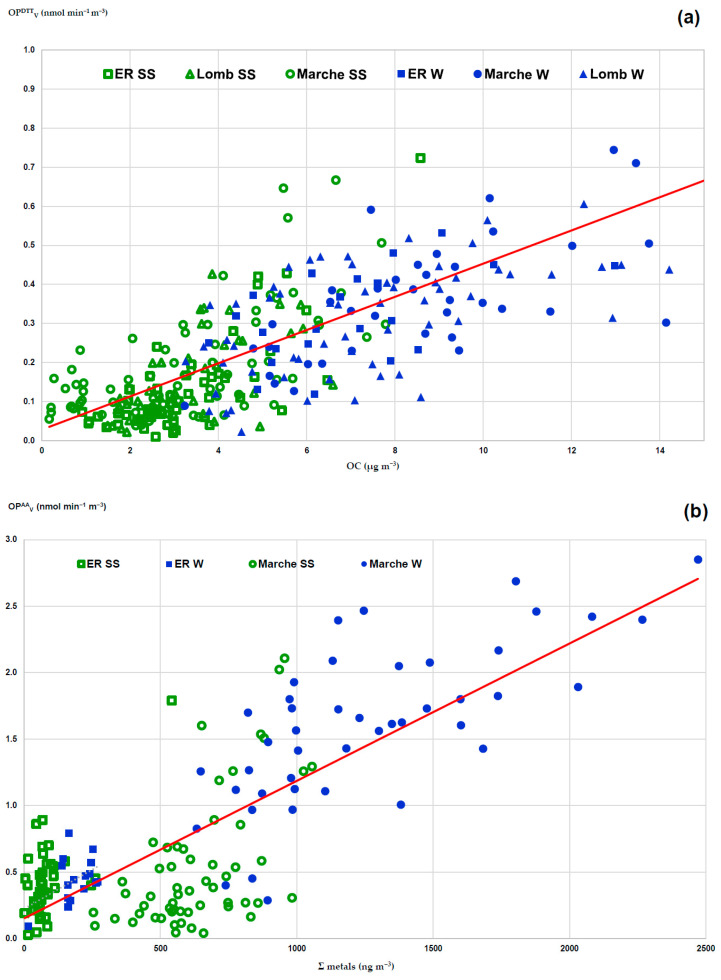
Relationship of the volume-normalized OP_V_ responses with concentration of emission tracers for filters collected at various sites across Po Valley in different seasons. (**a**) dependence of the OP^DTT^_V_ activity on OC concentration; (**b**): dependence of the OP^AA^_V_ activity on Σ Metals concentration. Squares indicate samples collected in Emilia Romagna sites (Bologna and S. Pietro), circles at MI_Marche and triangles at other sites in Lombardia region. Blue and green symbols describe winter and spring–summer (SS) samples, respectively.

To give a comprehensive insight into the contribution of the main emission sources driving the PM oxidative properties, their possible associations were investigated with the concentrations of OC, as tracers of carbonaceous emissions, and Σ Metals, accounting for vehicle emissions (Table 1 and Table 4) [12,15,30,32,33,37,46,47,48]. The major variations in OP^DTT^_V_ activity may be explained by the change in OC concentration, as supported by the discrete linear correlation (R^2^ = 0.645, *p* < 0.001, solid line in Figure 4a) found for a total of 305 samples, whatever the sampling site and season (Table 1 and Table 4). Higher OC and OP^DTT^_V_ values were shown by winter data, among the seasons (full blue symbols in the figure), with a small discrimination among the sites (squares, circles and triangles in the figure). The OP^AA^_V_ activity was found mostly associated with the total metal concentration, as supported by the discrete linear correlation (R^2^ = 0.641, *p* < 0.001, full line in Figure 4b) computed for 240 samples. In both seasons, higher metal levels and OP^AA^_V_ values were shown at MI_Marche, compared with the Bologna sites (circles and squares in the figure, respectively).

### 3.4. Impact of Lockdown Restrictions on Air Quality

The effect of the pandemic limitations was estimated by comparison with the baseline conditions commonly present at the MI_Marche site. To describe the meteorological and air quality parameters in 2019, we chose the ambient temperature and the solar radiation intensity, to account for the main meteorological variations, and the air levels of NO_2_ to describe automobile emissions, particularly from diesel engines [2,3,5,19,21,49]. The choice was also based on the data availability on the ARPA Lombardy and ER datasets [44,50]. The suitability of the NO_2_ concentration to represent the impact of vehicular emissions, was confirmed by its significant correlation (at *p* ≤ 0.01) with most of the investigated markers, mainly transition metals related to traffic source, as well as with the OP^AA^_V_ values (Pearson’s correlation coefficients in Table 2). For each lockdown period, the reference scenario was described by the mean values of the data retrieved from the database in the corresponding time span in 2019 (Table 4). These data were the basis to estimate the exclusive impact of the adopted lockdown strategies on air quality, independent of seasonal variation. A similar approach has recently been used in other studies in the Lombardia area [2,4,8,18,21,49]. By comparing the temperature and solar radiation values in the corresponding time spans, we can observe a general homogeneity between the two years and all over the region, with close value ranges in each investigated season (Table 1 and Table 4). Concerning air quality, during each season, the NO_2_ levels always showed higher levels at MI_Marche than at the other sites, even during the lockdown periods. This is consistent with the high impact from heavy traffic at this urban traffic station [5,19,27,28,29,41,48]. The lockdown impact on air NO_2_ levels was evaluated as the bias (expressed in %) of the concentrations in the PL1, FL and PL2 periods compared with those in the corresponding time spans in 2019. We computed a NO_2_ decrease to 82% during PL1, to 71% during FL and then a weak recovery to 77%, during PL2. These values may be used to estimate the reduction in the traffic emissions, although they must be confirmed with experimental data from real ambient samples.

### 3.5. Impact of Lockdown Restrictions on PM Oxidative Properties

A straight comparison of the OP^AA^_V_ and OP^DTT^_V_ responses measured in 2020 was performed with the data of other sites in the Po Valley in 2019, to represent the baseline scenario (Table 4). To evaluate the exclusive lockdown impact by disregarding the seasonal changes, the corresponding time intervals were chosen for data comparison, namely the FL period with spring in 2019 and the PL2 period with early summer in 2019 (Table 3 and Table 4). A general insight into the whole dataset showed that both the OP responses measured at MI_Marche were in general higher than those at other sites. However, some reductions can be observed in the periods impacted by the pandemic restrictions, compared with values at the urban sites in 2019. During FL, the OP^AA^_V_ values at MI_Marche showed a decrease to 81% and to 86% with respect to Bologna and MI Pascal, respectively, and during PL2 to 84% compared with MI Pascal. Otherwise, the OP^DTT^_V_ data at MI_Marche showed similar and even higher values than those at the urban sites in 2019, except for a drop to 40% with respect to MI-Pascal during PL2.

## 4. Discussion

First, some common trends can be singled out for the OP responses measured at all sites across the Po Valley. This suggests that all the investigated PM_2.5_ samples share common sources of redox-active components responsible for their oxidative properties, regardless of locations of urban/rural site and winter/SS season. For each PM_2.5_ sample, the AA assay produced higher OP responses in comparison with the DTT method. Such differences have also been observed in several works, specifically concerning urban areas [13,14,15,16,17,33,41]. This has been associated with the chemical composition of PM_2.5_ particles at sites strongly impacted by vehicle traffic emissions, since they typically contain high concentrations of traffic-related metals (e.g., Cu, Fe, Mn), towards which the AA assay is more responsive than the DTT method [30,31,34,43,48].

Another common characteristic is the strong seasonality of air pollution observed across the whole region, as described by variations in OP values and the concentration of PM mass and chemical components. This is clearly evident from a visual inspection of the data measured at MI_Marche during the whole study period, form winter to early summer, with a similar trend of both AA and DTT responses, as well as for PM_2.5_ and PM_10_ masses and metals concentrations (Figure 2 and Figure 3). In line with the findings from several studies in the area, this trend has been explained by the combination of meteorological factors associated with the impact of emission sources [25,26,27,28,29,45,51,52,53]. In the Po Valley, stable weather conditions and weak atmospheric mixing are prevalent in winter, which facilitate pollutant accumulation in the lower layers of the atmosphere and favor secondary processes, as described by the high contribution of ammonium nitrate, up to 60% in PM_2.5_. In contrast, in summer higher wind speed generates a broader mixing layer, which favors the pollutants’ dispersion in the atmosphere. In addition, large emissions are present in winter, mainly from extensive domestic biomass burning for residential heating, generating high levels of PM carbonaceous species. The increased level of these components has been found mainly responsible for the high OP values measured in winter by the authors and others in northern Italy [12,31,41,53,54,55] and in Europe [16,30,32,33,34,48,56,57].

Inside such similar trends, a deeper insight into the data singles out some differences among the sites, even if not always statistically significant, likely due to the wide variability within each monitoring campaign. We can observe a general increase in OP values at the urban sites in Milan and Bologna, compared with the rural background of Schivenoglia and San Pietro. Of the two OP metrics, the OP^AA^ responses were more scattered, providing higher discrimination between the sampling sites in comparison with the more homogenous OP^DTT^_V_ values. This is specifically evident in winter, when the OP^AA^_V_ responses at MI_Marche were nearly double or triple in comparison with those at the urban Bologna and MI_Pascal sites and even five times higher than those at the rural Schivenoglia. Consistently, also in the warm seasons the OP values showed differences among the sites, although reduced, decreasing according to the following order:

Marche > MI_Pascal ~ Bologna > S. Pietro ~ Schivenoglia.

This reflects differences in the strength of the PM_2.5_ sources across the region, with larger emissions from vehicular or other anthropogenic sources at the Milan and Bologna sites in comparison with the rural background sites [26,28,29,40].

The concentrations of PM_2.5_ components at MI_Marche and in other sites in the Po Valley showed consistent values with the literature data, reflecting the unique composition and emission sources of PM_2.5_ in urban and industrial areas in Italy [5,12,18,24,25,26,28,53,56] and in heavy traffic sites all over Europe [22,32,46,51,57,58]. High levels of OC and levoglucosan are emitted in winter from traffic and biomass-burning sources, which also directly emit total sulfur [5,27,40,45,53]. Vehicular traffic is the main source of the redox-active metals—Cr, Mn, Fe, Cu, Zn and Pb—in both exhaust and non-exhaust particles [5,37,41,45,48,52,57,59,60]. By comparing the metal concentrations in the PM size fractions simultaneously collected, we can observe nearly double levels in PM_10—_Σ metals 2.7 µg m^−3^ and nearly 1 µg m^−3^ in winter and warm seasons, respectively_,_ compared to the fine PM—Σ metals around 1 µg m^−3^ and 0.6 µg m^−3^ in winter and warm seasons, respectively (Table 1). This is in line with the metal size distribution found in the literature, where metals have been found commonly accumulated in the coarse fraction, as mainly emitted from tire/brake abrasion and fugitive re-suspended road dust [24,31,41,48,56,59]. Among the seasons, increased values were observed in winter compared with the warm season, following the seasonal trend of PM_2.5_ mass level. Among the sites, higher levels were shown in urban than in rural sites, with larger differences for Σ metals than for OC concentrations, reflecting the larger impact of vehicle emissions, mainly at the MI_Marche site, since it is the most impacted by heavy duty truck traffic [31,34,41,48].

Based on the correlation analysis on the MI_Marche dataset (Table 2), we can identify the species significantly correlated with OP_V_ to give a comprehensive insight into the emission sources determining the PM oxidative properties. They are emissions from biomass burning, traced by OC and levoglucosan, and vehicular traffic, described by the redox-active metals—Cr, Mn, Fe, Cu, Zn and Pb. The obtained results single out the specific sensitivity of the two OP assays, as described by the association of OP^DTT^_V_ activity with OC concentration and OP^AA^_V_ values with the total metal concentration [30,31,34,36,41,48,57]. These observations are in good agreement with the earlier studies across the Po Valley, including the metropolitan area of Milan [12,28,31,52,53]. These correlations hold even when considering the larger dataset, including other sites in the Po Valley (Figure 4), thus highlighting the general homogeneity of the sources of PM_2.5_ redox-active in the investigated area.

To give a general insight into the changes of the PM oxidative properties associated with the lockdown strategies, the measured values were compared with the literature data on fine PM, although only a few studies are available. The OP^AA^ and OP^DTT^ responses obtained in the pre-L period are within the typical ranges observed for fine particles collected at other urban and industrial areas in central and northern Italy [12,16,31,59], and in Europe [15,30,46,48,56,57]. Conversely, the responses obtained during the PL1, FL and PL2 periods showed reductions in PM_2.5_ OP when compared with those measured during spring/summer in urban sites and cities in Italy [15,31,59] and across Europe during the warm season [15,22,30,32,34,46,48,56].

Relevant information could be retrieved by the straight comparison of both the OP^AA^_V_ and OP^DTT^_V_ responses measured in our laboratory (Table 3) [41], since they captured complementary information on the different PM redox-active components [30,31,33,43,46,56,58]. In particular, the AA assay, being specifically sensitive to the traffic-related heavy metals, mainly measured the variation associated with the restrictions in road traffic [37,48,59]. They were drastically imposed by the Italian government shutdowns, which limited road and non-road transport to 48–60% [3,5,18,20]. The consequent decrease in the air NO_2_ levels observed in this study are in line with the findings from other authors in Milan, although there are small discrepancies mainly due to the choice of the time span [2,4,19,21,49]. Compared with such reductions, the OP^AA^_V_ responses showed a lower decrease to nearly 81–86% in the FL and PL2 periods, respective to the year 2019. This reduction was also lower than that to 53% during FL found in our previous work on PM_10_ filters at the MI_Pascal site [23]. Such a significantly weaker effect on PM_2.5_ than on PM_10_ OP can be explained by considering the prevalent accumulation in the coarse fraction of the traffic-related metals, which mainly drive the OP^AA^ response. Thus, the traffic limitation had a larger impact on metal concentration in the PM_10_ than in the PM_2.5_ particles, the effects of meteorological variations being constant. The time evolution of the metal concentrations in both fractions singled out that such an accumulation effect was the highest during the PreL and Pl1 periods and decreased during the following FL and PL2 periods, showing similar metal levels in the two size fractions, when emissions from traffic decreased (Figure 3).

The OP^DTT^_V_ responses presented similar levels in both 2020 and 2019 years. Based on the strong association of OP^DTT^ reactivity with biomass burning sources, this trend may be explained by the low reduction in the PM_2.5_ carbonaceous fraction, mostly emitted from domestic residential heating and garden bonfires [53,57]. Such activities remained nearly constant and even enhanced during the lockdown, since most of the population was confined at home, following the Italian Government imposition [2,8,20,21,24]. Thus, the current study suggests that variation in the local source of vehicular traffic could provide a small contribution to the OP^DTT^ of fine PM. Our findings are in line with the other available studies in Athens and China, which reported that OP^DTT^ levels were not reduced during the lockdown period, despite the significant reduction in the traffic-related pollutants [6,22]. However, our results on the OP^DTT^_V_ variation at the MI_Marche site showed a slightly lower impact of the pandemic restrictions in comparison with the data reported by Altuwayjiri at the MI Pascal site in similar periods [2].

## 5. Conclusions

This work provides general insights into the spatial distributions of the PM_2.5_ oxidative properties on a regional scale across the Po Valley. The obtained results confirmed the alarming air quality of the highly populated area, indicating high human toxicological risk. The study took advantage of combining OP responses from both AA and DTT assays, giving specific information on PM_2.5_ emission sources. The spatial distribution of OP data across the region singled out elevated OP^AA^ at the locations largely impacted by traffic. This is the case of the MI_Marche site in the metropolitan area of Milan, which showed high oxidative toxicity and pollutant concentrations, also during the pandemic restrictions. Because of the severe lockdown limitations in road traffic, a small reduction was observed in oxidative toxicity to nearly 81–86% of the OP values measured in normal conditions across the region.

As a final remark, the results emphasize the relevance of acellular assays in measuring OP. This parameter integrates toxicological properties and the chemical composition of the PM to provide valuable information on the air pollution status and human and ecological risk. This may be the basis to design environmental policies, green actions and strategies leading to healthier living environments.

## Figures and Tables

**Table 1:** The OP responses measured with AA and DTT assays, concentrations of PM_2.5_ mass and its chemical components, air levels of atmospheric pollutants (nitrogen dioxide, NO_2_ and black carbon, BC), meteorological parameters (ambient temperature and solar radiation). Reported values are means ± standard deviation values computed for each lockdown period. * indicates means with significant differences (Student *t*-test. *p* ≤ 0.05) between the periods. BLQ = below quantification limit. Italic values indicate estimated values from data experimentally measured on PM_10_ samples.

	PreL January–25 February	PL1 26 February–24 March	FL 25 March–4 May	PL2 5–18 May
OP^AA^_V_ (nmol min^−1^m^−3^)	1.72 * ± 0.78 888	0.72 ± 0.68	0.38 ± 0.37	0.43 ± 0.40
OP^DTT^_V_ (nmol min^−1^m^−3^)	0.43 * ± 0.22	0.21 ± 0.13	0.25 ± 0.20	0.12 ± 0.05
OP^AA^_m_ (nmol min^−1^µg^−1^)	0.041 ± 0.030	0.026 ± 0.024	0.023 ± 0.021	0.024 ± 0.052
OP^DTT^_m_ (nmol min^−1^µg^−1^)	0.008 ± 0.003	0.008 ± 0.006	0.015 ± 0.008	0.011 ± 0.008
PM_2.5_ (µg m^−3^)	51.46 * ± 18.43	25.2 ± 8.9	16.5 ± 7.42	10.6 ± 3.18
OC (µg m^−3^)	*9.99* * ± *4.11*	*4.62* ± *1.45*	*3.90* ± *1.43*	*0.77* ± *0.41*
Levo (µg m^−3^)	*0.66* * ± *1.54*	*0.17* ± *0.09*	*0.09* ± *0.05*	*BLQ*
P (ng m^−3^)	12.2 * ± 4.4	5.40 ± 2.47	3.01 ± 1.27	BLQ
S (µg m^−3^)	1046 * ± 511	767 ± 334	1047 ± 515	913 ± 215
Cl (ng m^−3^)	785 * ± 536	74.2 ± 85.3	108 ± 193	127 ± 193
Al (ng m^−3^)	0.00	0.00	0.00	0.00
Si (ng m^−3^)	332 ± 177	251 ± 74	437 ± 387	287 ± 172
K (ng m^−3^)	524 * ± 250	219 ± 75	196 ± 90	152 ± 58
Ca (ng m^−3^)	349 ± 186	255.06	342 ± 552	215 ± 116
Ti (ng m^−3^)	21.8 ± 9.4	13.8 ± 4.3	18.8 ± 14.9	14.5 ± 7.4
V (ng m^−3^)	BLQ	BLQ	BLQ	BLQ
Cr (ng m^−3^)	8.63 * ± 2.53	4.88 ± 1.23	5.08 ± 0.99	5.63 ± 0.32
Mn (ng m^−3^)	13.7 * ± 5.9	5.30 ± 2.82	5.49 ± 3.65	5.28 ± 2.74
Fe (ng m^−3^)	1115 * ± 400	527 ± 226	576 ± 226	556 ± 68
Ni (ng m^−3^)	8.86 ± 1.48	7.71 ± 0.40	7.89 ± 0.37	BLQ
Cu (ng m^−3^)	42.5 * ± 13.7	18.9 ± 7.5	15.7 ± 2.5	17.7 ± 3.2
Zn (ng m^−3^)	88.1 * ± 35.7	40.4 ± 21.9	24.0 ± 19.9	29.2 ± 34.2
Br (ng m^−3^)	12.3 * ± 6.4	7.65 ± 3.88	6.66 ± 3.08	5.14 ± 1.92
Rb (ng m^−3^)	2.53 ± 1.12	1.34 ± 0.45	7.26 ± 4.45	7.15 ± 2.29
Pb (ng m^−3^)	38.7 * ± 12.8	10.8 ± 8.9	2.07 ± 4.60	2.03 ± 5.21
Σ metals_2.5_ (µg m^−3^)	1.29 * ± 0.49	0.62 ± 0.26	0.60 ± 0.22	0.61 ± 0.10
PM_10_ (µg m^−3^)	56.4 * ± 20.0	27.8 ± 9.7	21.5 ± 9.8	15.6 ± 4.1
Σ metals_10_ (µg m^−3^)	2.71 * ± 0.91	1.13 ± 0.36	0.81 ± 0.32	0.67 ± 0.15
NO_2_ (µg m^−3^)	78.71 ± 20.88	52.98 ± 10.76	40.83 ± 11.9	30.43 ± 8.94
BC (µg m^−3^)	4.84 * ± 2.34	1.71 ± 0.68	1.34 ± 0.84	0.52 ± 0.16
Temperature (°C)	6.89 ± 2.55	10.07 ± 2.87	12.72 ± 3.79	19.72 ± 2.38
Rad (W m^−2^)	107.6 ± 97.2	508.2 ± 213.1	618.7 ± 175.9	732.3 ± 238.4

**Table 2:** Pearson’s correlation coefficients of the OP^AA^_V_ and OP^DTT^_V_ responses and air levels of NO_2_ with the concentrations of PM_2.5_ and PM_10_ mass, PM_2.5_ chemical components and air levels of NO_2_. Bold values indicate significant correlation at *p* < 0.01 level (*n* = 144).

	OP^AA^_V_	OP^DTT^_V_	NO_2_
OP^AA^_V_ (nmol min^−1^m^−3^)	1.00	**0.65**	**0.65**
OP^DTT^_V_ (nmol min^−1^m^−3^)	**0.65**	1.00	0.15
PM_2.5_ (µg m^−3^)	**0.73**	**0.74**	**0.63**
OC (µg m^−3^)	**0.62**	**0.63**	**0.60**
Levo (µg m^−3^)	**0.72**	**0.78**	**0.68**
S (µg m^−3^)	0.11	0.40	0.07
Cl (ng m^−3^)	**0.62**	**0.62**	**0.52**
Al (ng m^−3^)	−0.20	−0.05	0.04
Si (ng m^−3^)	−0.11	−0.11	0.04
K (ng m^−3^)	**0.70**	**0.81**	**0.61**
Ca (ng m^−3^)	0.00	0.10	**0.41**
Ti (ng m^−3^)	0.19	0.27	0.35
Cr (ng m^−3^)	**0.62**	0.49	**0.51**
Mn (ng m^−3^)	**0.59**	**0.66**	**0.62**
Fe (ng m^−3^)	**0.61**	**0.59**	**0.61**
Ni (ng m^−3^)	0.45	0.24	0.16
Cu (ng m^−3^)	**0.70**	**0.63**	**0.70**
Zn (ng m^−3^)	**0.68**	**0.69**	**0.60**
Br (ng m^−3^)	**0.56**	**0.69**	**0.45**
Rb (ng m^−3^)	−0.23	−0.04	−0.27
Pb (ng m^−3^)	**0.60**	**0.60**	**0.61**
Σ metals_2.5_ (µg m^−3^)	**0.63**	**0.58**	**0.59**
PM_10_ (µg m^−3^)	**0.71**	**0.72**	**0.59**
NO_2_ (µg m^−3^)	**0.57**	**0.46**	1.00

**Table 3:** Overview of samples analyzed in this study: sampling period, site location and number of PM_2.5_ collected filters for each sampling campaign.

Sampling Period	Sampling Site	Sample Number	Abbreviation
Winter			
2 January–11 February 2020	Milan_Pascal	41	MI_Pascal
2 January–25 February 2020	Schivenoglia	55	Schivenoglia
4–25 February 2020	Bologna	22	Bologna
Spring			
10 March–2 April 2018	Bologna	30	Bologna
16 March–9 April 2018	S Pietro Capofiume	30	S Pietro
20–28 April 2019	Schivenoglia	9	Schivenoglia
20–28 April 2019	Milan_Pascal	9	MI_Pascal
Early Summer			
1 May–30 June 2019	Bologna	61	Bologna
8–16 June 2019	Schivenoglia	9	Schivenoglia
8–16 June 2019	Milan_Pascal	9	MI_Pascal

**Table 4:** The OP responses measured with AA and DTT assays, concentrations of PM_2.5_ mass and their chemical components, air levels of nitrogen dioxide, NO_2_, meteorological parameters (ambient temperature and solar radiation). Reported values are means ± standard deviation values computed for each sampling campaign described in Table 3. * indicate means with significant differences (Student *t*-test at *p* ≤ 0.05) between the periods. BLQ = below quantification limit. Italic values indicate estimated values from data experimentally measured on PM_10_ samples.

Winter		**Bologna**	**MI_Pascal**	**Schivenoglia**		
OP^AA^_V_ (nmol min^−1^m^−3^)		0.82 ± 0.61	1.02 ± 0.28	0.73 ± 0.15		
OP^DTT^_V_ (nmol min^−1^m^−3^)		0.29 ± 0.13	0.58 ± 0.15	0.53 ± 0.12		
OP^AA^_m_ (nmol min^−1^µg^−1^)		0.022 ± 0.018	0.013 ± 0.008	0.011 ± 0.008		
OP^DTT^_m_ (nmol min^−1^µg^−1^)		0.012 ± 0.009	0.007 ± 0.003	0.007 ± 0.003		
PM_2.5_ (µg m^−3^)		34.40 ± 16.18	46.11 ± 17.39	40.59 ± 18.80		
OC (µg m^−3^)		6.65 ± 2.46	*8.55* ± *2.91*	*6.60* ± *1.98*		
Levo (µg m^−3^)		0.57 ± 0.33	*0.76* ± *0.38*	*0.59* ± *0.39*		
Σ metals_2.5_ (µg m^−3^)		209.7 ± 120.0				
NO_2_ (µg m^−3^)		48.99 ± 8.48	52.70 ± 12.05	31.17 ± 10.31		
Temperature (°C)		9.75 ± 1.98	5.70 ± 2.79	5.70 ± 2.77		
Rad (W m^−2^)		113.9 ± 30.0	82.39 ± 34.72	82.21 ± 43.72		
PM_10_ (µg m^−3^)				52.15 ± 20.38		
Spring	**S Pietro**	**Bologna**	**MI_Pascal**	**Schivenoglia**	**MI_Marche Feb–March 2019**	**MI_Marche** **March–May 2019**
OP^AA^_V_ (nmol min^−1^m^−3^)	0.30 ± 0.23	0.47 ± 0.04	0.44 ± 0.41	0.21 ± 0.16		
OP^DTT^_V_ (nmol min^−1^m^−3^)	0.08 ± 0.04	0.27 ± 0.02	0.15 ± 0.08	0.08 ± 0.08		
OP^AA^_m_ (nmol min^−1^µg^−1^)	0.021 ± 0.0155	0.038 ± 0.017	0.033 ± 0.027	0.018 ± 0.017		
OP^DTT^_m_ (nmol min^−1^µg^−1^)	0.005 ± 0.004	0.029 ± 0.010	0.011 ± 0.005	0.006 ± 0.05		
PM_2.5_ (µg m^−3^)	13.63 ± 3.51	13.34 ± 2.44	12.59 ± 3.86	15.04 ± 10.15		
OC (µg m^−3^)	3.51 ± 1.36	3.99 ± 1.60	*4.01* ± *0.95*	*2.57* ± *0.79*		
Levo (µg m^−3^)	0.11 ± 0.07	0.12 ± 0.06	*0.04* ± *0.01*			
Σ metals_2.5_ (µg m^−3^)	30.7 ± 16.01	132.7 ± 93.0				
NO_2_ (µg m^−3^)	7.38 ± 4.09	25.46 ± 10.39	13.95 ± 7.44	11.89 ± 5.37	64.49 ± 15.2	57.17 ± 12.3
Temperature (°C)	9.74 ± 2.93	11.46 ± 3.39	15.86 ± 1.89	19.52 ± 2.72	12.82 ± 2.19	13.79 ± 3.78
Rad (W m^−2^)	165.3 ± 79.2	150.7 ± 69.9	223.4 ± 73.8	215.7 ± 81.5	171.1 ± 80.2	184.4 ± 92.4
PM_10_ (µg m^−3^)	35.7 ± 21.92	56.39 * ± 20.02	27.36 ± 9.96	21.93 ± 9.75		
Early summer		**Bologna**	**MI_Pascal**	**Schivenoglia**	**MI_Marche** **5–18 May 2019**	
OP^AA^_V_ (nmol min^−1^m^−3^)		0.36 ± 0.22	0.51 ± 0.38	0.04 ± 0.03		
OP^DTT^_V_ (nmol min^−1^m^−3^)		0.07 ± 0.03	0.31 ± 0.04	0.11 ± 0.06		
OP^AA^_m_ (nmol min^−1^µg^−1^)		0.039 ± 0.024	0.036 ± 0.022	0.003 ± 0.002		
OP^DTT^_m_ (nmol min^−1^µg^−1^)		0.008 ± 0.005	0.025 ± 0.006	0.007 ± 0.003		
PM_2.5_ (µg m^−3^)		9.83 ± 3.72	12.92 ± 3.86	14.92 ± 4.89		
OC (µg m^−3^)		2.44 ± 0.91	*3.87* ± *0.99*	*2.09* ± *0.56*		
Levo (µg m^−3^)		0.027 ± 0.012				
Σ metals_2.5_ (µg m^−3^)		55.9 ± 39.7				
NO_2_ (µg m^−3^)		11.71 ± 0.53	12.48 ± 5.05	7.11 ± 2.82	40.15 ± 4.4	
Temperature (°C)		20.49 ± 5.87	19.18 ± 2.08	24.42 ± 1.51	20.49 ± 2.18	
Rad (W m^−2^)		241.3 ± 89.1	238.1 ± 18.2	301.5 ± 44.3	239.8 ± 79.2	
PM_10_ (µg m^−3^)						

## Data Availability

The data presented in this study are available on request from the corresponding author.
